# Synthesis and Structural Evolution of Zn–Ni ZIF Materials: From Cubic to Lamellar Frameworks

**DOI:** 10.1021/acsomega.6c02492

**Published:** 2026-06-08

**Authors:** Iasmin Soares de Lima, Gabriel Alves da Silva, Lais Gomes Sanchez, Gabriel Iago dos Santos, Gilbert Bannach, Luiz G. Possato

**Affiliations:** † Department of Physics and Meteorology, School of Sciences, 28108 São Paulo State University (Unesp), Av. Eng. Luiz Edmundo Carrijo Coube 14-01, Bauru, São Paulo 17033-360, Brazil; ‡ Department of Chemistry, School of Sciences, 28108 São Paulo State University (Unesp), Av. Eng. Luiz Edmundo Carrijo Coube 14-01, Bauru, São Paulo 17033-360, Brazil

## Abstract

Understanding metal-dependent structural phase transitions in zeolitic imidazolate frameworks (ZIFs) remains a key challenge for the rational design of functional materials, particularly in controlling the transformation between distinct polymorphs such as ZIF-8 and ZIF-L. Despite recent advances, the role of transition-metal incorporation in directing phase evolution and tuning physicochemical properties is still not fully understood. This study reports the synthesis and structural evolution of bimetallic Zn_1–*x*_Ni_*x*_ (*x* = 0–0.8) zeolitic imidazolate frameworks prepared via a rapid methanolic precipitation route at room temperature. The results demonstrate that Ni incorporation drives a composition-dependent phase transition from the cubic ZIF-8 framework to the lamellar ZIF-L structure, revealing a controllable structural evolution in Zn–Ni ZIF systems. The effects of Ni incorporation on crystal structure, morphology, porosity, and thermal stability were systematically investigated by XRD, Raman spectroscopy, SEM/EDS, N_2_ physisorption, and TGA analyses. XRD and Rietveld refinement indicate that low Ni incorporation (*x* ≤ 0.2) preserves the ZIF-8 topology, while higher Ni contents (*x* ≥ 0.4) induce lattice strain, peak broadening, and the emergence of the ZIF-L phase. Raman spectroscopy reveals increased structural disorder and the coexistence of different coordination environments. Physisorption results show a gradual decrease in surface area and microporosity, while thermal analysis indicates that Ni incorporation modulates framework stability. CO_2_ adsorption results highlight the influence of phase evolution and morphology on adsorption performance. Overall, these findings demonstrate that Ni acts as a structural modulator and phase-directing agent, enabling controlled tuning of the ZIF-8 to ZIF-L transformation, with potential implications for the design of advanced materials for catalysis and gas adsorption.

## Graphical abstract

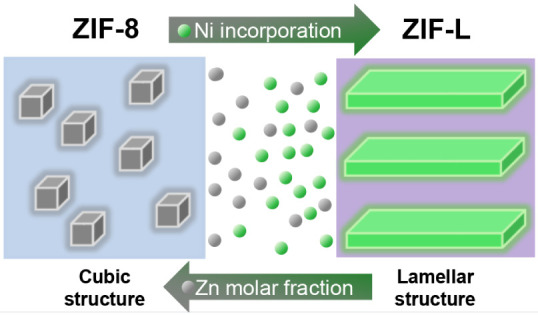

## 1 Introduction

Recent efforts in heterogeneous catalysis have increasingly emphasized the development of materials with high turnover frequencies via the rational design of isolated active sites or improved adsorption properties. In this context, metal–organic frameworks (MOFs) have attracted considerable attention due to their porous crystalline nature, consisting of metal nodes coordinated to organic linkers that form cage-like architectures with exceptionally high internal surface areas. Despite these advantages, their application in thermocatalysis is often constrained by limited thermal stability.^1–5^ Within this class of materials, zeolitic imidazolate frameworks (ZIFs) stand out as a subclass combining enhanced chemical and thermal robustness. Their imidazolate linkers, which contain nitrogen donor atoms, act as Lewis bases and promote the adsorption and activation of acidic molecules such as CO_2_.^6–8^ As a result, ZIFs bridge key features of conventional MOFs and zeolites, offering high crystallinity, tunable porosity, and improved structural stability.^1^ Structurally, ZIFs are formed by tetrahedrally coordinated metal centers, commonly, but not exclusively, Zn^2+^ or Co^2+^, linked by azolate-type ligands, particularly imidazolate derivatives, forming frameworks with zeolite-like topologies and enhanced chemical and thermal.^9,10^ The characteristic Im-M-Im bond angle (∼145°) closely resembles the Si–O–Si angle found in zeolites, which contributes to their enhanced framework stability and porosity.^11^

Among the most representative ZIF structures, ZIF-8 (Zn­(2-methylimidazolate)_2_) exhibits a sodalite (SOD) topology characterized by large cavities (∼11.6 Å) interconnected through narrow pore apertures (∼3.4 Å). Its cobalt analogue, ZIF-67, is isostructural with ZIF-8, sharing the same SOD topology, whereas ZIF-L is a layered two-dimensional polymorph with a distinct framework structure. Despite these differences in long-range topology and dimensionality, these materials exhibit similar local coordination environments, consisting of tetrahedrally coordinated metal centers linked by imidazolate ligands, which ultimately influence their physicochemical properties and potential applications.^12,13^ Recent studies have increasingly focused on the structural and functional modulation of ZIF materials by incorporating secondary metal centers. In particular, bimetallic ZIF systems have been shown to exhibit modified coordination environments, electronic structures, and adsorption properties, arising from the distinct preferences of different metal ions.^10,14^ These effects are closely related to the ability of transition metals to alter nucleation and crystal growth pathways, ultimately influencing phase formation, morphology, and pore accessibility. In addition, recent reviews have emphasized that the structural behavior of ZIFs is strongly governed by the interplay between coordination geometry, linker flexibility, and synthesis conditions, which collectively determine the stability and functionality of the resulting frameworks.^10,15^ One of the main applications of ZIFs structures is in heterogeneous catalysis, where pure or functionalized ZIF-8 acts as a catalyst or support for reactions such as Knoevenagel condensation, transesterification for biodiesel production, and hydrogenation.^16^ Another relevant application is the separation and adsorption of gases, for which ZIF-8-based materials are effective at purifying complex mixtures, including CO_2_.^7^

Due to their tunable pore structures, large surface areas, and versatile metal centers, ZIFs have been widely investigated for diverse applications, including gas adsorption and separation, catalysis, drug delivery, electrocatalysis, and as precursors for functional nanostructured materials after pyrolysis.^6,7,17^ In particular, the structural flexibility of ZIFs allows fine-tuning of their physicochemical properties through metal substitution or ligand modification, enabling the design of bimetallic frameworks such as Ni-ZIFs and Co-ZIFs with enhanced catalytic and adsorption performance.

Bimetallic ZIFs have recently gained attention because incorporating secondary transition-metal ions enables precise modulation of the framework’s coordination environment and electronic structure.^18^ In ZIF-8, partial substitution of Zn^2+^ by other divalent cations such as Ni^2+^ or Co^2+^ has been reported to introduce mixed metal-imidazolate nodes that tune metal–ligand bond strength, charge distribution, and defect density, ultimately influencing nucleation and crystal growth mechanisms.^18^

Introducing Ni^2+^ into the ZIF-8 lattice is therefore an effective strategy to tailor the metal-imidazolate bonding strength; however, the role of Ni in directing phase evolution between different ZIF polymorphs remains poorly understood. Ni^2+^ exhibits a stronger ligand field and a preference for octahedral coordination, which can significantly influence crystal growth kinetics, framework topology, and defect formation. Consequently, Ni-doped ZIF-8 systems provide an ideal platform to explore structure–property relationships and to design bimetallic ZIF precursors with improved catalytic performance.^19^ In this work, we demonstrate that Ni incorporation can drive a controlled structural transition from cubic ZIF-8 to lamellar ZIF-L.

## 2 Materials and Methods

### 2.1 Synthesis of the Materials

The catalysts were synthesized by a rapid precipitation method in a methanolic solution at room temperature.^8,20^ Zinc nitrate hexahydrate (Zn­(NO_3_)_2_·6H_2_O, Sigma-Aldrich, 98%) was first dissolved in methanol (40 mL) under magnetic stirring for 5 min. Separately, 2-methylimidazole (Sigma-Aldrich, 99%) and nickel nitrate hexahydrate (Ni­(NO_3_)_2_·6H_2_O, Sigma-Aldrich, 97%) were each dissolved in methanol (40 mL) and stirred for 5 min. The two solutions were then mixed, maintaining different Zn:Ni molar ratios to evaluate the structural effects of Ni presence (Table 1).

**Table 1:** Mass of Reactants Weighted for Each Molar Ratio Synthesis

Zn:Ni	2-Methylimidazole (g)	Zn(NO_3_)_2_·6H_2_O (g)	Ni(NO_3_)_2_·6H_2_O (g)
Zn_1.0_Ni_0.0_	0.526	0.810	-
Zn_0.8_Ni_0.2_	0.526	0.648	0.158
Zn_0.6_Ni_0.4_	0.526	0.486	0.316
Zn_0.4_Ni_0.6_	0.526	0.324	0.475
Zn_0.2_Ni_0.8_	0.526	0.162	0.633

A pure Zn sample (Zn_1.0_Ni_0.0_) was first prepared as a reference, followed by bimetallic samples with nominal Zn:Ni molar ratios of 0.8:0.2, 0.6:0.4, 0.4:0.6, and 0.2:0.8, hereafter labeled Zn_0.8_Ni_0.2_, Zn_0.6_Ni_0.4_, Zn_0.4_Ni_0.6_, and Zn_0.2_Ni_0.8_, respectively. The resulting mixtures were allowed to react for 24 h at room temperature without agitation. The precipitates were then separated by centrifugation, washed twice with 20 mL of methanol, centrifuged again at 3000 rpm for 10 min, and finally dried at 50 °C for 24 h. A pure Ni sample was also prepared following the same procedure; however, no solid precipitate was observed.

### 2.2 Characterization

The crystalline phases in the calcined samples were analyzed by X-ray diffraction (XRD) using a Rigaku MiniFlex 600 diffractometer with CuKα radiation (λ = 1.5406 Å) operating at 40 kV and 15 mA. Data were collected in the 2θ range from 5 to 90°, with a scan step interval of 0.05°. The Rietveld refinement of the X-ray diffraction patterns was performed using the GSAS-II software.^21,22^ The initial structural model for ZIF-8 was taken from the literature, and, when applicable, a two-phase model including ZIF-L was considered.^10^ Peak profiles were modeled using a pseudo-Voigt function, and the background was fitted using a shifted Chebyshev polynomial (6th order). The refinement strategy was carried out sequentially, starting with the scale factor and zero shift, followed by lattice parameters, peak profile parameters (U, V, W), and microstructural parameters (crystallite size and microstrain). Atomic positions and occupancies were kept fixed due to the limited sensitivity of laboratory XRD data to these parameters in MOF-type structures. No preferred orientation correction was applied. The quality of the refinements was evaluated using the weighted profile R-factor (R_wp_), and the results were interpreted with caution, considering the structural complexity and possible disorder of the samples.^22^

Raman scattering spectra were collected for the catalysts before and after the reaction using an i-Raman Plus 532 H portable spectrometer with a high quantum efficiency CCD array. The spectra were acquired with a fiber optic probe and excitation wavelength of 532 nm (30 mW, nominal), 60 s of integration time, and an average of 3 spectra was obtained.

The stability of the samples was analyzed using Thermogravimetry (TG), Differential Thermal Analysis (DTA), and Derivative Thermogravimetry (DTG) techniques to assess thermal decomposition. Under oxidizing atmosphere, the analyses were performed using an STA 449 F3 instrument (Netzsch) in the temperature range of 25–1000 °C (10 °C min^–1^), using 10 mg of the sample, an open α-Al_2_O_3_ crucible, and a dry air atmosphere (70 mL min^–1^).

Nitrogen adsorption–desorption isotherms were measured at 77 K on an Anton Paar Autosorb 6100 system. Before measurements, the samples (ca. 10 mg) were degassed under vacuum at 120 °C for 12 h. The apparent Brunauer–Emmett–Teller (BET) surface areas were determined from nitrogen physisorption isotherms by selecting the fitting region according to the Rouquerol criteria, ensuring the appropriate application of the BET model. It should be noted that, for microporous materials such as ZIFs exhibiting type I isotherms, the BET model does not yield a true probe-accessible surface area; instead, it provides an apparent surface area that serves as a useful comparative adsorbent fingerprint.^23^ The density functional theory (DFT) calculations for the pore size distribution curves were done with the native NovaWin 11.03 software using the N_2_ at 77 K on carbon, slit pore, nonlocal density functional theory (NLDFT) equilibrium model for nitrogen. In addition, none of the present DFT kernels for N_2_ on carbon reflect the surface properties of a ZIF material. The numbers can be used for the comparison of similar materials, but must not be taken as exact values for the pore sizes or surface areas of ZIFs. In the absence of MOF-specific kernels, the N_2_ on carbon kernels with different pore types is frequently used to study the surface properties of the ZIFs.^23^

The morphology and microstructure of the samples were examined by scanning electron microscopy (SEM) using a Hitachi TM3030 tabletop microscope equipped with a Bruker XFlash 430 energy-dispersive X-ray spectroscopy (EDS) detector. The accelerating voltage was set to 15 kV. The samples were analyzed without a conductive coating under high-vacuum mode. EDS spectra and elemental mappings were acquired to determine the qualitative distribution of Zn, Ni, C, and N within the crystals. The EDS data were used to verify the presence of both Zn and Ni in the bimetallic samples and to evaluate the homogeneity of metal distribution across the particles. For each sample, 16 spectra were acquired in distinct regions of the crystals.

The CO_2_ adsorption experiments were carried out in a fixed-bed glass tubular reactor operated under continuous gas flow. Before each run, the system was purged with N_2_ (45 mL·min^–1^) at 120 °C for 30 min to remove atmospheric contaminants. Subsequently, a known amount of sample (100 mg) was loaded into the reactor, supported by glass wool. Blank experiments were also conducted under identical conditions: (i) with the reactor bypassed and (ii) with the empty reactor, to quantify baseline fluctuations and the intrinsic response of the system. The adsorption tests were performed by feeding a gas mixture of 27 mL·min^–1^ N_2_ and 3 mL·min^–1^ CO_2_ (10% CO_2_ in N_2_, total flow rate of 30 mL·min^–1^), controlled by mass flow controllers. The reactor outlet was connected to a gas chromatograph equipped with a thermal conductivity detector (GC/TCD), which continuously monitored the CO_2_ concentration as a function of time. Breakthrough profiles were recorded for 40 min.

## 3 Results and Discussion

Figure 1a shows the X-ray diffraction (XRD) patterns of the Zn_1–*x*_Ni_*x*_ (*x* = 0–0.8) samples, while Figure 1b highlights the magnified region around the (011) reflection. The Zn_1.0_Ni_0.0_, Zn_0.8_Ni_0.2_, Zn_0.6_Ni_0.4_, and Zn_0.4_Ni_0.6_ samples exhibit the characteristic diffraction peaks of the ZIF-8 framework, which can be indexed to the SOD topology, including the planes (011), (002), (112), (022), (013), and (222), confirming the preservation of the sodalite-type topology in the presence of Ni during synthesis. For clarity, the main diffraction peaks corresponding to the ZIF-L phase have also been indexed in Figure 1a, particularly for the Zn_0.2_Ni_0.8_ sample, allowing a direct distinction between the reflections of ZIF-8 (SOD topology) and those associated with the layered ZIF-L structure. Reference patterns of ZIF-8 and ZIF-L are provided in Figure S1 for comparison. Interestingly, the introduction of a small amount of Ni (Zn_0.8_Ni_0.2_) leads to a marked increase in diffraction peak intensity compared to pure ZIF-8 (Zn_1.0_Ni_0.0_). This enhancement may be associated with improved crystallinity and/or increased crystal size, as supported by SEM analysis (Figure 2), where the Zn_0.8_Ni_0.2_ sample exhibits well-defined and larger crystallites with smoother facets. Nickel incorporation at low concentrations can act as a crystallization promoter, modifying nucleation kinetics and favoring slower crystal growth, which is consistent with the formation of more ordered domains.^24^ Additionally, the partial replacement of Zn^2+^ by Ni^2+^ may slightly increase the electron density contrast between the framework and the imidazolate ligands, enhancing X-ray scattering efficiency.

**Figure 1:**
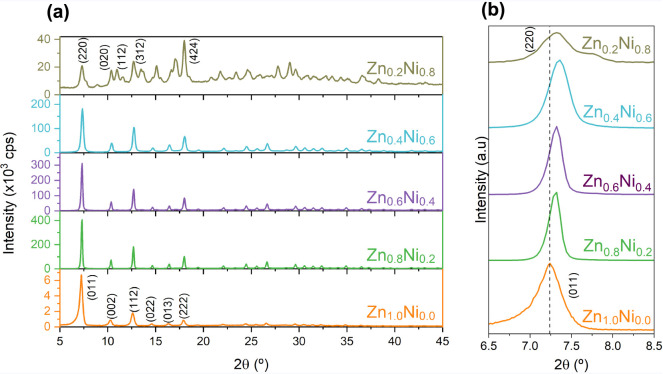
(a) X-ray diffraction (XRD) patterns of Zn_1–*x*_Ni*_x_* (*x* = 0–0.8) samples, highlighting the characteristic reflections of the ZIF-8 (SOD topology) and the emergence of reflections consistent with the ZIF-L phase, particularly for the Zn_0.2_Ni_0.8_ sample. (b) Magnified region showing the (011) reflection of ZIF-8 and the (220) reflection associated with ZIF-L, evidencing a systematic shift toward higher 2θ values with increasing Ni content, indicative of lattice contraction.

**Figure 2:**
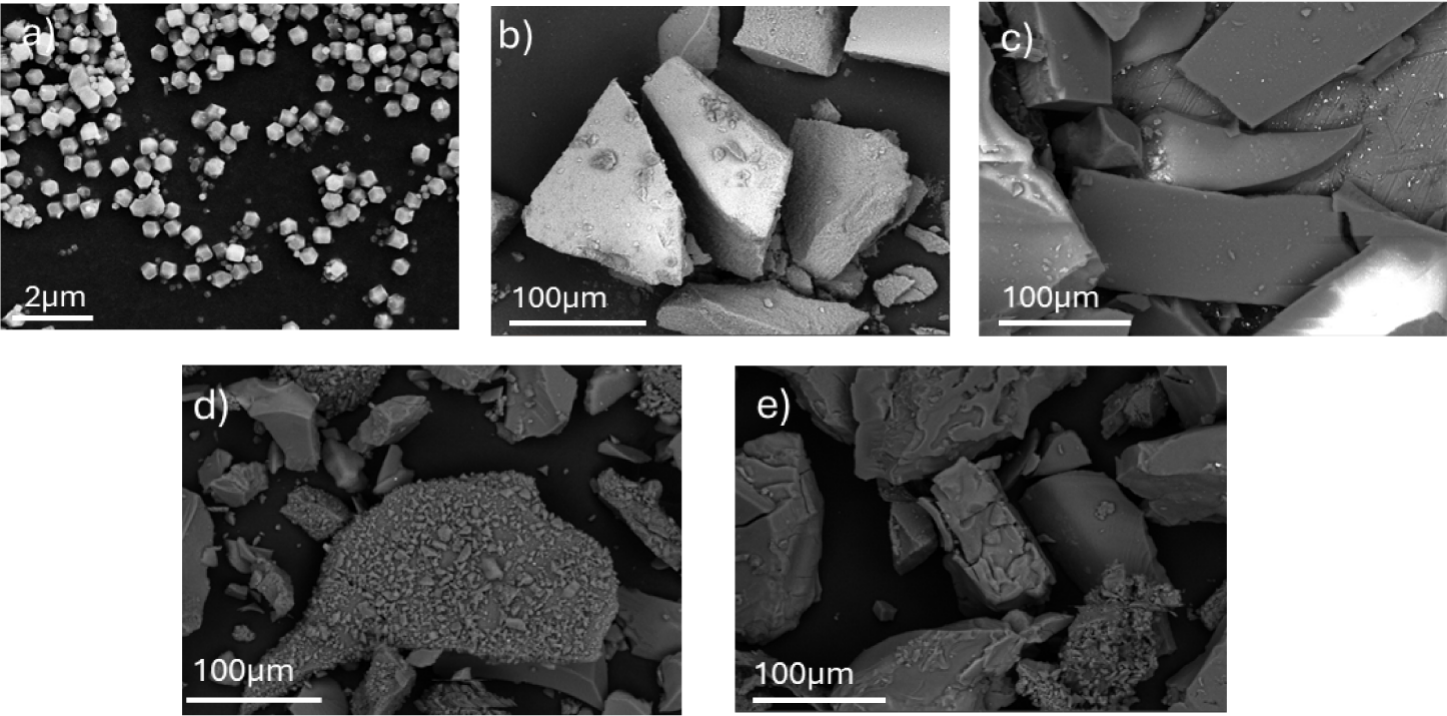
SEM micrographs of Zn_1–*x*_Ni*_x_* (*x* = 0–0.8) samples: (a) Zn_1.0_Ni_0.0_, (b) Zn_0.8_Ni_0.2_, (c) Zn_0.6_Ni_0.4_, (d) Zn_0.4_Ni_0.6_, and (e) Zn_0.2_Ni_0.8_.

As Ni content increases further (*x* ≥ 0.4), the overall intensity of the reflections decreases, and the peaks broaden, indicating reduced long-range order and a progressive loss of crystallinity. The Zn_0.2_Ni_0.8_ sample shows broad, poorly resolved peaks and altered intensity ratios, suggesting a structural transition or phase coexistence. These features, along with the lamellar morphology, are consistent with the formation of ZIF-L, a layered polymorph with a distinct crystal structure and diffraction pattern compared to ZIF-8, which often forms under room-temperature methanolic synthesis conditions, as observed for the Zn_0.2_Ni_0.8_ sample.^18^ The magnified low-angle region (Figure 1b) also reveals a systematic shift of the (011) reflection toward higher 2θ values as Ni content increases, confirming lattice contraction caused by the smaller ionic radius of Ni^2+^ (0.69 Å, tetrahedral coordination) compared to Zn^2+^ (0.74 Å, tetrahedral coordination).^25^ The smaller Ni^2+^ ions can locally contract the M–N bonds, increasing framework rigidity and altering the relative growth rates of crystallographic planes. At higher Ni concentrations, the stronger ligand field and octahedral tendency of Ni^2+^ may contribute to the disruption of the sodalite-type topology of ZIF-8, potentially promoting anisotropic growth along the ab-plane and stabilizing the lamellar ZIF-L structure. Under Ni-excess conditions, Ni^2+^ is expected to exhibit a tendency toward octahedral coordination geometry (ionic radius = 0.83 Å), which induces local expansion and structural rearrangement of the framework, being consistent with the formation of the lamellar ZIF-L phase. These combined geometric and electronic effects explain the observed morphological transition from cubic to lamellar crystals and the associated reduction in long-range order detected by XRD.

Rietveld refinement provided quantitative evidence of the structural evolution induced by nickel incorporation (Tables 2 and 3 and Figure S2). The lattice parameter of the ZIF-8 phase increased slightly from 16.66 Å (Zn_1.0_Ni_0.0_) to 17.04 Å (Zn_0.2_Ni_0.8_), consistent with the presence of lattice strain and partial relaxation within the framework. The crystallite size varied irregularly with Ni content, reaching a maximum for Zn_0.8_Ni_0.2_ (46 nm), in agreement with the larger and more ordered lamellar crystals observed by SEM, and then decreasing sharply for higher Ni loadings. This reduction to about 0.04 μm in Zn_0.2_Ni_0.8_, together with the substantial increase in microstrain (ε ≈ 30 × 10^–3^), indicates strong structural distortion and loss of long-range order. In particular, the Zn_1.0_Ni_0.0_ sample exhibited a relatively higher Rwp value (15.2%), which reflects the intrinsic limitations of refining MOF-type structures with large unit cells, possible local disorder, and microstructural effects such as preferred orientation. This observation highlights that even for single-phase systems, the refinement quality may be limited, and therefore, the derived parameters should not be overinterpreted.

**Table 2:** Crystallographic Parameters Obtained by Rietveld Refinement

Sample	Lattice parameter a (Å)	Crystallite size (nm)	ε (10^–3^)	R_wp_ (%)
Zn_1.0_Ni_0.0_	16.6639 ± 0.0001	21	0.240	15.2
Zn_0.8_Ni_0.2_	17.0292 ± 0.0001	46	0.812	9.8
Zn_0.6_Ni_0.4_	17.0312 ± 0.0001	40	1.846	11.1
Zn_0.4_Ni_0.6_	17.0355 ± 0.0001	27	3.767	8.5
Zn_0.2_Ni_0.8_ ^a^	17.0404 ± 0.0001	16	29.983	2.7

a The sample contains 3 wt % of the ZIF-8 phase.

**Table 3:** Crystallographic Parameters Obtained by Rietveld Refinement of Zn_0.2_Ni_0.8_ ZIF-L Phase (97wt%)^a^,^b^

a (Å)	b (Å)	c (Å)	Crystallite size (nm)	ε (10^–6^)
24.1183	17.0334	19.7180	20	0.5

a R_wp_ = 2.7.

b The sample contains 3 wt % of the ZIF-8 phase.

For the Zn_0.2_Ni_0.8_ sample (Table 3), a two-phase refinement provided the best fit (R_wp_ = 2.7%), revealing the coexistence of approximately 97 wt % of a ZIF-L phase and 3 wt % residual ZIF-8. The orthorhombic unit cell parameters of this ZIF-L-type structure (a = 24.12 Å, b = 17.03 Å, c = 19.72 Å) confirm a transition from the cubic ZIF-8 topology to a lamellar configuration. This phase transformation explains both the pronounced broadening of the diffraction peaks and the morphological change to thin plates detected by SEM.

The pure ZIF-8 sample (Zn_1.0_Ni_0.0_, Figure 2a) displays well-defined cubic crystals with smooth facets and uniform size distribution, consistent with typical ZIF-8 morphology reported in the literature.^25^ The introduction of a small amount of Ni (Zn_0.8_Ni_0.2_) leads to a notable increase in crystal size and a morphological transition from cubic to slightly lamellar morphology. These large, well-developed crystals suggest that nickel incorporation at low levels slows down the nucleation rate while promoting anisotropic growth, allowing the formation of more ordered crystallites.^18,26^ This observation correlates well with the enhanced peak intensities and sharper reflections observed in XRD (Figure 1), confirming that the material exhibits greater crystallinity and a preferred growth orientation.

At intermediate compositions (Zn_0.6_Ni_0.4_ and Zn_0.4_Ni_0.6_), the crystals become smaller and more irregular, with partially aggregated particles and rougher surfaces. The reduction in crystal size and appearance of surface defects indicates increased disorder and strain within the framework, in agreement with the XRD peak broadening and the onset of structural distortion induced by Ni presence. For the Ni-rich sample (Zn_0.2_Ni_0.8_), the morphology changes drastically to thin lamellar aggregates, forming stacked or sheet-like structures. This two-dimensional morphology is characteristic of the ZIF-L polymorph, which is known to form under room-temperature, methanolic conditions and exhibits a layered structure rather than the three-dimensional cubic framework of ZIF-8. The coexistence of lamellar and cubic morphologies in the Ni-rich materials supports the hypothesis of a mixed ZIF-8/ZIF-L phase system or the presence of highly defective ZIF-8 domains that have partially reorganized into a lamellar configuration.

Besides, the pronounced morphological change observed even at low Ni contents arises from the intrinsic difference between Zn^2+^ and Ni^2+^ coordination chemistry. Nickel ions have a smaller ionic radius and a stronger ligand field, forming more stable Ni–N bonds and preferentially coordinating in octahedral environments.^24^ Their presence alters the balance between nucleation and crystal growth, promoting slower and anisotropic growth that produces larger, lamellar morphology crystals. As the Ni fraction increases, the stronger Ni–N interactions and the reduced preference for tetrahedral coordination destabilize the sodalite-type ZIF-8 network. Consequently, the framework reorganizes into the lamellar ZIF-L structure, in which metal centers exhibit mixed tetrahedral-octahedral coordination, and the growth proceeds preferentially along the *ab* plane. This structural transition explains the morphological evolution and the progressive reduction in long-range order observed in XRD and SEM.

Figure 3a shows the Raman spectra of Zn_1–*x*_Ni_*x*_ (*x* = 0–0.8). All samples retain the characteristic bands of the 2-methylimidazolate (mIm) linker, indicating preservation of the imidazolate framework. With increasing Ni content, the ν­(M–N) band in the low-wavenumber region shifts toward lower wavenumbers (red shift) and gradually broadens; a similar trend, albeit weaker, is seen for ring-related modes in the 680–1000 cm^–1^ window (Figure 3b).

**Figure 3:**
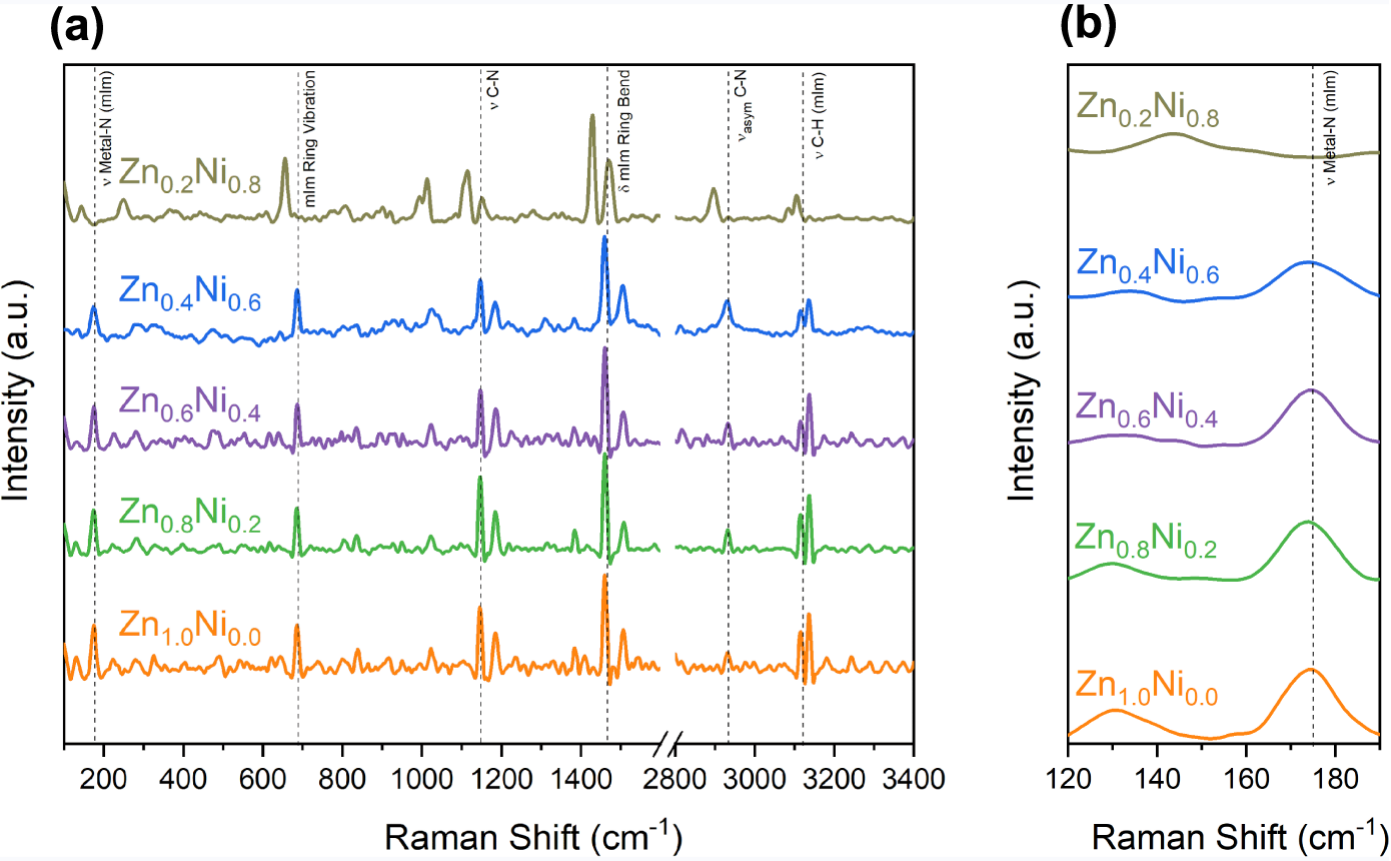
Raman spectra of Zn_1–*x*_Ni*_x_* (*x* = 0–0.8) samples. (a) Full spectra highlighting the main vibrational modes of the 2-methylimidazolate ligand. (b) Expanded view of the ν­(M–N) region showing a gradual red shift and broadening with increasing Ni content, consistent with lattice distortion and lamellar (ZIF-L-like) domains.

For Zn_0.8_Ni_0.2_ the band intensity increases markedly while the peak position remains essentially unchanged or slightly downshifted; this intensity gain is attributed to larger, better-ordered crystals and preferred orientation of the emerging lamellar morphology. From *x* ≥ 0.4, the red shift becomes evident, and in the Ni-rich sample (Zn_0.8_Ni_0.2_), the shift and broadening are pronounced, consistent with lower symmetry, increased structural disorder, and lamellar (ZIF-L-like) domains, which soften the average metal–nitrogen and ring vibrational force constants. These Raman features align with XRD (loss of crystallinity and lattice strain) and SEM (transition from cubic to lamellar morphologies).

All materials display typical type I isotherms according to the IUPAC classification, confirming the predominantly microporous nature of the structures (Figure 4).^27^ The sharp uptake at low relative pressures (p/p_0_ < 0.1) is characteristic of permanent microporosity associated with the sodalite-type topology of ZIF-8. However, subtle variations in the isotherm profiles and hysteresis loops are observed as the Ni content increases, indicating the progressive modification of the pore network and the emergence of intercrystalline mesoporosity related to the development of lamellar morphologies. The pure Zn_1.0_Ni_0.0_ sample exhibits a high surface area of 1298 m^2^·g^–1^, a total pore volume of 0.54 cm^3^·g^–1^, and an average pore width of ∼1.43 nm, consistent with the values reported for highly crystalline ZIF-8 frameworks.^7^ The selection of the fitting region was performed according to the Rouquerol consistency criteria. Representative BET and Rouquerol plots for all samples are provided in the Supporting Information (Figure S3). Such high apparent BET values may arise from interparticle porosity and from the use of apparent BET analysis derived from type-I isotherms. The isotherm shows a steep rise at low relative pressures followed by a long plateau up to p/p_0_ ≈ 0.9, confirming a uniform microporous system. This behavior agrees with the XRD results, indicating a cubic phase, and the SEM images show well-defined cubic crystals.

**Figure 4:**
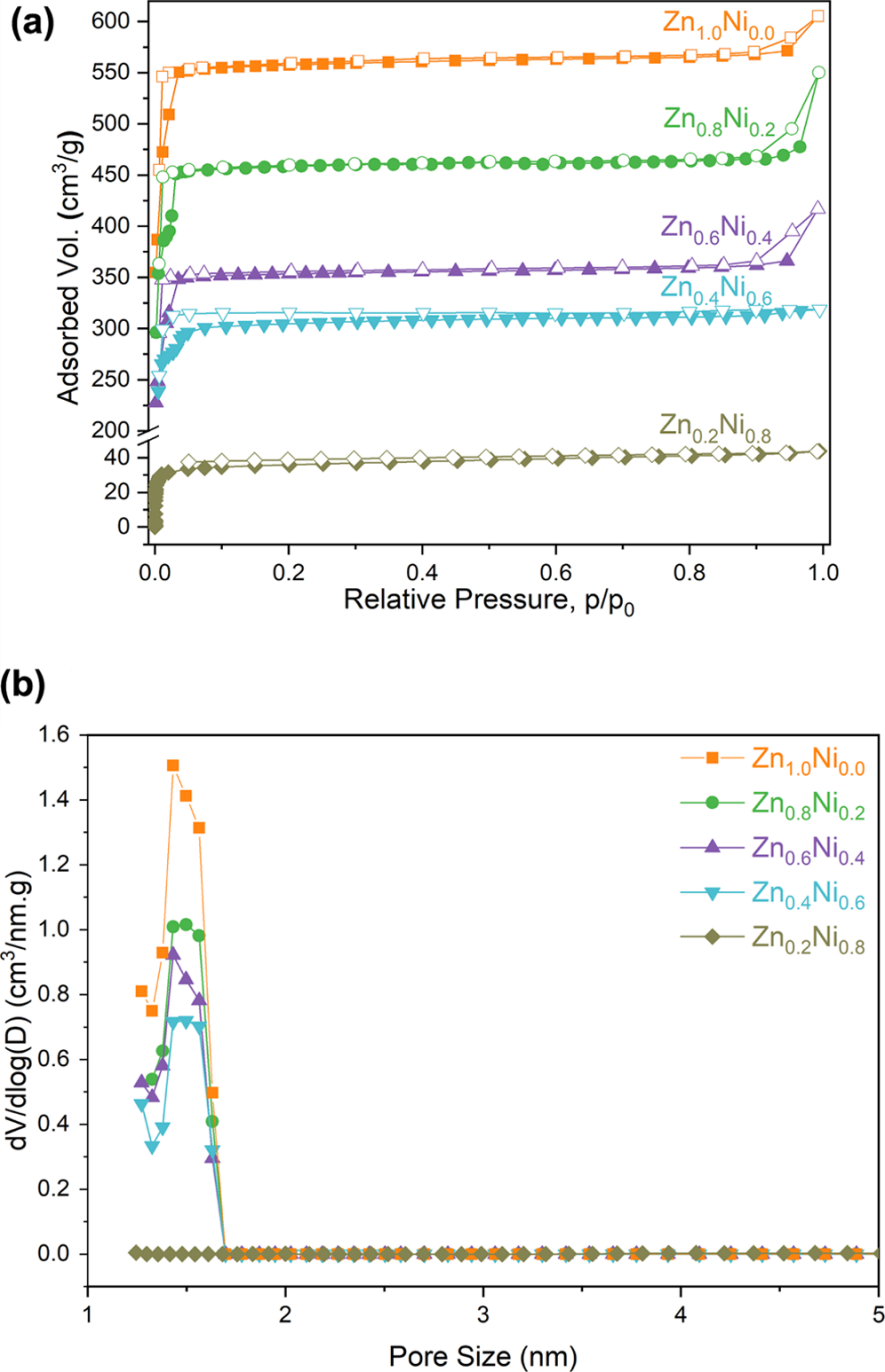
(a) N_2_ adsorption–desorption isotherms (filled points correspond to nitrogen adsorption and empty points to desorption), and (b) pore-size distributions of the samples.

At low Ni concentration (Zn_0.8_Ni_0.2_), the surface area was 2023 m^2^·g^–1,^ and the total pore volume is 0.55 cm^3^·g^–1^, while the mean pore size remains close to 1.50 nm. Although the surface area slightly decreases compared to pure ZIF-8, the increase in total pore volume and the smoother hysteresis region suggest a partial opening of the pore system and the formation of small mesopores between the larger, lamellar morphology crystals observed by SEM. These textural changes, together with the sharper XRD peaks and higher thermal stability seen in TGA, indicate that low Ni content during synthesis appears to act as a crystallization promoter, leading to larger, better-organized ZIF-8 domains with anisotropic growth.

For the Zn_0.6_Ni_0.4_ sample, the material exhibits a surface area of 1001 m^2^·g^–1^ and a total pore volume of 0.59 cm^3^·g^–1^, with an average pore size of ∼1.43 nm. A small hysteresis loop appears at high relative pressures (p/p_0_ > 0.8), suggesting the presence of interconnected mesopores and the coexistence of cubic and lamellar domains. The combination of both micro- and mesoporous features reflects the generation of structural defects and interlamellar voids as the Ni content increases. The Zn_0.4_Ni_0.6_ sample showed a pronounced reduction in surface area (1038 m^2^·g^–1^) and pore volume (0.48 cm^3^·g^–1^), while maintaining a modal pore width around 1.50 nm. The isotherm presents a more gradual slope and a mild hysteresis at higher p/p_0_. The decrease in surface area and micropore volume confirms the partial collapse of the three-dimensional framework as the material transitions to a two-dimensional lamellar configuration. The interlayer voids in ZIF-L contribute to secondary mesoporosity, but their accessibility is lower than that of the intrinsic micropores in ZIF-8.

Zn_0.2_Ni_0.8_ displays a markedly different adsorption behavior. This composition exhibits a significantly reduced apparent BET surface area of 111 m^2^ g^–1^, accompanied by a very low nitrogen uptake across the entire relative pressure range. The pore size distribution becomes poorly defined, indicating the loss of accessible microporosity. This behavior is consistent with the structural transition toward a lamellar ZIF-L-like phase, as evidenced by XRD and SEM analyses, in which the layered arrangement and stronger interlayer interactions limit N_2_ diffusion at 77 K. Therefore, the drastic reduction in surface area for Zn_0.2_Ni_0.8_ is not merely a consequence of Ni presence but reflects a fundamental change in framework topology and porosity, resulting in a material with restricted gas-accessible surface area.

The TG/DTG-DTA curves are shown in Figure 5. For ZIF-based materials, the major mass-loss event is typically associated with framework decomposition at temperatures above ∼450 °C. The temperature ranges (θ), mass loss values (Δ*m*), peak temperatures (*T*_*p*_), temperature of the maximum degradation rate (*T_MDR_*), and maximum degradation rate (*MDR*) associated with each mass loss stage of the samples are shown in Table 4. The sample Zn_1.0_Ni_0.0_ (the system without nickel) exhibited three steps of mass loss, characteristic of typical ZIF-8 materials.^28^ The first step was associated with the removal of occluded methanol molecules or excess linkers. The second step corresponds to the main thermal decomposition of the ZIF-8 framework, occurring in the ∼450–550 °C range, associated with the breakdown of the imidazolate linkers and collapse of the structure. The subsequent mass loss is attributed to the oxidation of residual carbonaceous species, resulting in exothermic events in the DTA curve.^28^

**Figure 5:**
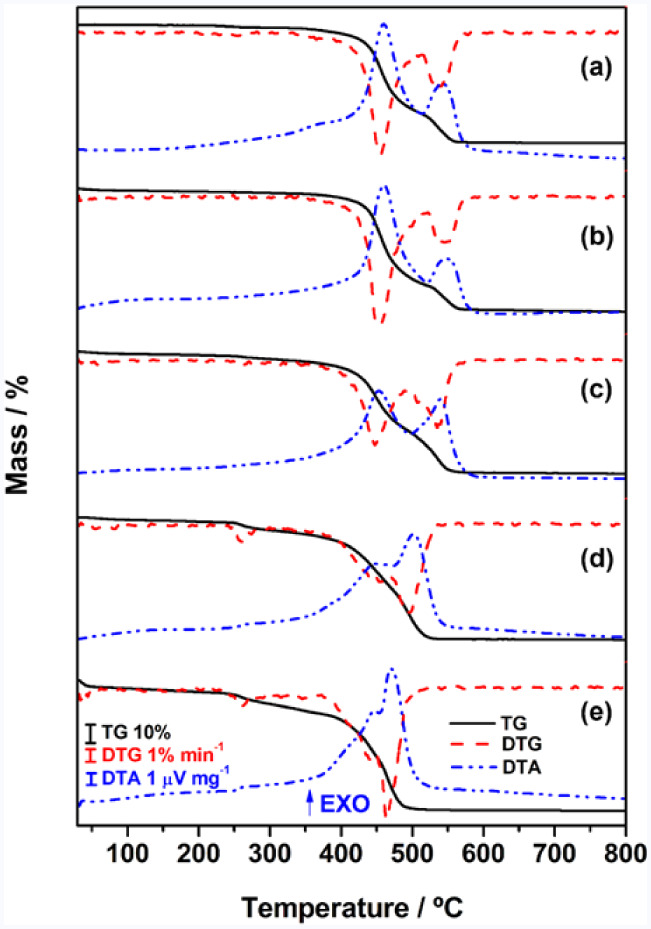
TG/DTG-DTA curves for (a) Zn_1.0_Ni_0.0_, (b) Zn_0.8_Ni_0.2_, (c) Zn_0.6_Ni_0.4_, (d) Zn_0.4_Ni_0.6_, and (e) Zn_0.2_Ni_0.8_.

**Table 4:** Mass Loss Values (Δ*m*), Temperature Ranges (θ), Peak Temperatures (*T_p_*), Temperature of the Maximum Degradation Rate (*T_MDR_*), and Maximum Degradation Rate (*MDR*) Associated with Each Mass Loss Step of the Complexes[Table-fn tbl4fn1]

		**Sample**				
Step		Zn_1.0_Ni_0.0_	Zn_0.8_Ni_0.2_	Zn_0.6_Ni_0.4_	Zn_0.4_Ni_0.6_	Zn_0.2_Ni_0.8_
1	Δ*m*/%	1.1	0.9	0.9	1.3	3.8
	θ/°C	225.3–307.4	30.0–83.3	30.0–81.2	30.0–84.0	30.0–60.3
	*T*_*p*_/°C	—	—	—	—	—
	*T*_*MDR*_/°C	255	—	—	—	—
	*MDR*/% min^–1^	0.3	—	—	—	—
2	Δ*m*/%	47.3	0.8	1.4	1.6	2.94
	θ/°C	307.4–512.4	235.7–322.8	81.2–249.4	84.0–241.0	60.3–220.3
	*T*_*p*_/°C	369↑; 460↑	—	—	—	—
	*T*_*MDR*_/°C	456	—	—	—	—
	*MDR*/% min^*–*1^	9.7	—	—	—	—
3	Δ*m*/%	16.0	50.2	2.5	5.2	11.1
	θ/°C	512.4–589.1	322.8–517.6	249.4–332.6	241.0–325.5	220.3–363.4
	*T*_*p*_/°C	542↑	460↑	281↑	252↓; 271↑	250↓; 262↑
	*T*_*MDR*_/°C	531.0	456	259	261	259
	*MDR*/% min^*–*1^	4.4	10.2	0.5	1.4	1.5
4	Δ*m*/%	—	—	61.7	59	53.7
	θ/°C	—	—	332.6–602.0	325.5–575.9	363.4–556.3
	*T*_*p*_/°C	—	548↑	453↑; 539↑	447↑; 501↑	447↑; 470↑
	*T*_*MDR*_/°C	—	546	447; 507; 535	444; 454; 496	444; 464
	*MDR*/% min^*–*1^	—	3.7	6.8; 3.4; 5.41	4.4; 4.6; 7.1	5.9; 10.4
Residue	m/%	35.3	32.6	33.3	32.3	28.1

i ↑ Exothermic event; ↓ Endothermic event.

When nickel complexes are incorporated into the formulation, the thermal behavior starts to change, with all samples presenting four steps of mass loss. Except for the Zn_0.8_Ni_0.2_ sample, the other systems (Zn_0.6_Ni_0.4_, Zn0._4_Ni_0.6_, and Zn_0.2_Ni_0.8_) exhibited a similar profile, with the first and second mass-loss steps associated with the removal of solvent from the surface and solvent trapped within the material, respectively. Besides, the third mass-loss step is mainly associated with the thermal decomposition of the ZIF framework, which may overlap with the release of residual or weakly bound organic species. This process occurs over a broader temperature range compared to pure ZIF-8, reflecting the increased structural complexity of the Ni-containing systems.^29,30^ This interpretation is further supported by an endothermic event followed by an exothermic event on the DTA curve for the samples containing the highest amounts of nickel complexes (Zn_0.4_Ni_0.6_ and Zn_0.2_Ni_0.8_). Moreover, the increase in the amount of this material in the formulation led to an increase in the mass loss percentage at this step. Finally, the last mass-loss step was associated with the degradation of the zinc-complex ligands, which occurs in a single step but in a more complex manner, as evidenced by overlapping events in the DTG curve. Regarding sample Zn_0.8_Ni_0.2_, while the loss of trapped solvent was not observed, the same thermal events identified in the samples containing higher amounts of nickel complex were observed, as well as the thermal decomposition of the organic ligand in the zinc complex, followed by the oxidation of carbonaceous matter occurring in two steps of mass loss according to DTG curve (similar to Zn_1.0_Ni_0.0_).

As shown in Figure 5 and Table 4, the behavior of the DTG curve was clearly affected by the addition of the nickel complex to the formulation, mainly during the thermal decomposition step of the ligand in the zinc complex. Consequently, increasing the amount of nickel complex resulted in a reduction in the maximum degradation rate (MDR) values during this step when compared with pure ZIF-8 (9.7% min^–1^). In contrast, an increase in the MDR values of the final step was observed.

The residual mass observed at the end of all curves is associated with zinc and nickel oxides. Although a slight decrease is noted after the addition of the nickel complex to the formulation, overall, the incorporation of the ligands into the structure of the complexes does not appear to be compromised.

Point EDS analyses (Figure S4 and Table 5) complemented the morphological observations. Only Zn and C were detected in Zn_1.0_Ni_0.0_, and Ni remained below the detection limit in Zn_0.8_Ni_0.2_ and Zn_0.6_Ni_0.4_, indicating that the Ni concentration in individual crystals is very low or inhomogeneously distributed. In contrast, clear Ni signals were observed for Zn_0.4_Ni_0.6_ and Zn_0.2_Ni_0.8_, indicating the increasing presence of Ni within the material. Conversion of the detected Zn and Ni weight percentages into atomic ratios showed that the local Zn/Ni ratios are lower than the nominal values, demonstrating that Ni presence becomes more effective at higher nominal Ni loadings. Together with the structural and thermal data, these results confirm that Ni incorporation intensifies as the Ni content increases, promoting the development of lamellar ZIF-L-like domains containing mixed Zn/Ni coordination environments.

**Table 5:** Samples Composition Determined by XRD and EDS

Sample	Nominal Ni/Zn ratio	XRD determined Ni/Zn ratio	EDS Ni/Zn ratio	Comment
Zn_1.0_Ni_0.0_	0	0	0.00	Pure ZIF-8
Zn_0.8_Ni_0.2_	0.25	0	0.00	Ni below detection by EDS
Zn_0.6_Ni_0.4_	0.67	...	0.00	Ni below detection by EDS
Zn_0.4_Ni_0.6_	1.50	0.011	0.21	Clear Ni presence by EDS
Zn_0.2_Ni_0.8_ ^a^	4.00	...	0.45	Ni-rich lamellar phase

a The sample contains 3 wt % of the ZIF-8 phase.

It should be noted that EDS provides semiquantitative information and is primarily sensitive to the surface composition; therefore, the values reported here are used to confirm compositional trends rather than absolute metal contents.

As an additional effort to confirm the presence of nickel in the structure, Rietveld refinements were performed on the calcined samples (Figure 6 and Table 5). For the Zn_0.4_Ni_0.6_ composition, the refinement revealed the presence of approximately 1 wt % of crystalline NiO after calcination. The (200) plane of NiO is marked with “*” in Figure 6. This value corresponds to a Ni/Zn molar ratio of ∼0.011, indicating that only a minor fraction of nickel segregates as a detectable crystalline oxide phase. The low amount of NiO observed suggests that most of the nickel species are either highly dispersed, incorporated into the ZnO lattice as a solid solution, or present in an amorphous form below the detection limit of X-ray diffraction. Although limited in sensitivity, this result provides complementary evidence supporting the effective incorporation of Ni into the ZIF-derived structure, in agreement with the trends observed by TG, SEM/EDS, and structural analyses of the pristine frameworks.

**Figure 6:**
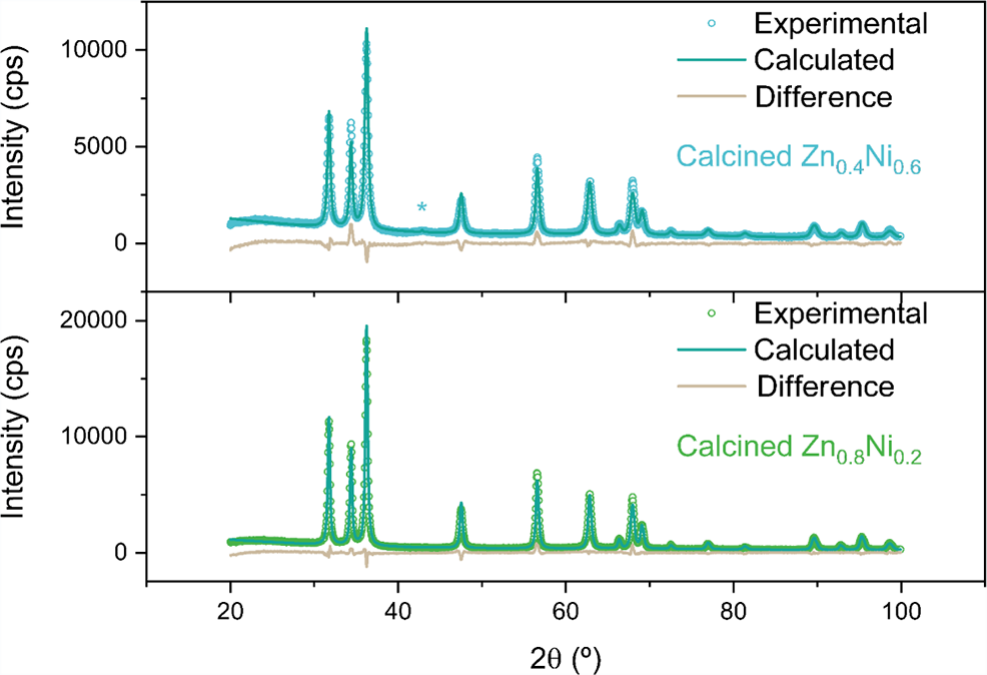
Rietveld refinement of the powder X-ray diffraction patterns of the calcined Zn_0.8_Ni_0.2_ and Zn_0.4_Ni_0.6_ samples. The asterisk (*) marks the reflection corresponding to the (200) plane of NiO.

Although advanced spectroscopic techniques such as XPS or EXAFS would provide more direct evidence of the local coordination environment of Ni, the combined structural, morphological, and thermal analyses clearly demonstrate that Ni strongly influences the crystallization behavior and phase evolution of the Zn–Ni ZIF system.

Based on the CO_2_ adsorption results (Figure 7) obtained for the Zn/Ni bimetallic ZIFs, a nonmonotonic dependence on the nominal Ni content is observed, reflecting the combined effects of structural evolution and changes in adsorption sites. Zn-rich samples (Zn_1.0_Ni_0.0_ to Zn_0.4_Ni_0.6_), which preserve a ZIF-8-type structure, display comparable adsorption capacities, indicating that partial Ni presence does not linearly enhance CO_2_ uptake. In this compositional range, adsorption is mainly governed by micropore accessibility and diffusion limitations, with Ni playing a secondary role by introducing structural disorder rather than creating significantly stronger adsorption sites.

**Figure 7:**
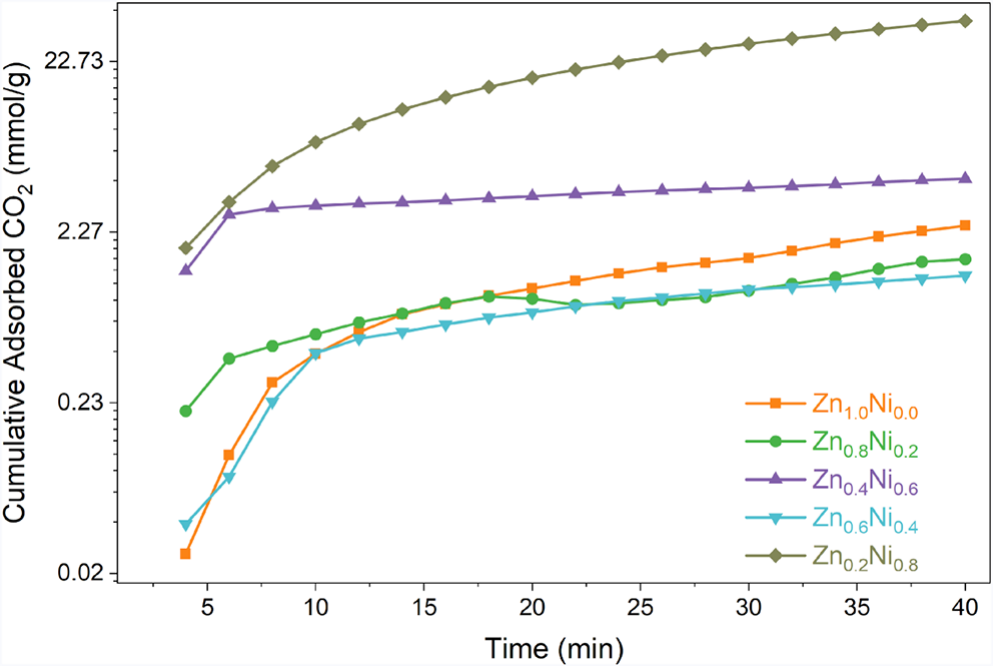
Cumulative CO_2_ adsorbed of samples Zn_1.0_Ni_0.0_, Zn_0.8_Ni_0.2_, Zn_0.6_Ni_0.4_, Zn0._4_Ni_0.6_, and Zn_0.2_Ni_0.8_.

In contrast, the Ni-rich Zn_0.2_Ni_0.8_ sample exhibits a markedly higher CO_2_ uptake, which is attributed to a structural transition toward a lamellar ZIF-L-like phase, as evidenced by XRD, SEM, and surface area analysis. This morphology provides enhanced external surface exposure and reduced diffusional resistance, enabling faster and higher CO_2_ adsorption. Additionally, the higher density of defects and heterogeneous coordination environments in Ni-rich systems may enhance CO_2_-framework interactions. Therefore, the CO_2_ capture performance of Zn–Ni ZIFs is controlled by the interplay between phase evolution, pore accessibility, defect density, and metal–ligand coordination environment, rather than by a simple increase in Ni content. The observed behavior highlights that intermediate compositions may suffer from partial loss of microporosity without sufficient gain in accessible adsorption sites, whereas Ni-rich compositions benefit from a distinct adsorption mechanism associated with the ZIF-L structure. The CO_2_ uptake values obtained under dynamic flow conditions should not be directly compared with equilibrium adsorption capacities reported for ZIF materials. The measurements reflect a combined contribution of adsorption kinetics and surface reactivity under continuous CO_2_ exposure, rather than equilibrium physisorption.

## 4 Conclusions

The systematic Ni presence during the ZIF-8 synthesis significantly influenced the structural, morphological, and thermal properties of the frameworks. At low Ni contents, Ni^2+^ acts as a crystallization promoter, enhancing crystal growth and producing larger, lamellar morphology crystals with higher crystallinity and thermal stability. Structural analyses confirmed that the sodalite-type ZIF-8 topology is preserved under these conditions. As the Ni concentration increases, however, the incorporation of Ni^2+^, with its smaller ionic radius and octahedral coordination tendency, induces lattice strain and structural disorder, driving the framework toward partial conversion into the lamellar ZIF-L phase. Nickel acts as a phase-directing agent that induces a controllable structural evolution from ZIF-8 to ZIF-L in Zn–Ni systems.

Rietveld refinements, Raman spectroscopy, and nitrogen physisorption consistently revealed a progressive transition from a highly ordered microporous cubic network to a less connected lamellar framework with mixed tetrahedral-octahedral coordination. The Ni-rich ZIF-L-like domains exhibit decreased surface area and reduced thermal stability, consistent with their lower structural connectivity. These results establish a clear structure-composition relationship in Ni-doped ZIF-8, highlighting the dual role of nickel as both a structural modulator and a phase-directing agent. More importantly, they demonstrate that CO_2_ uptake in bimetallic ZIFs is governed primarily by phase evolution and morphological features rather than by the nominal metal composition alone. This insight provides a rational basis for tailoring ZIF-based materials through controlled metal incorporation and phase engineering to achieve improved performance under dynamic CO_2_ adsorption conditions.

## Supplementary Material



## Notes

XRD reference patterns, Rietveld refinement profiles of Zn_1–*x*_Ni_*x*_ (*x* = 0–0.8), BET and Rouquerol plots, SEM micrograph, and corresponding EDS spectra (PDF)
