# Supplementary material for: Synthesis and Structural Evolution of Zn–Ni ZIF Materials: From Cubic to Lamellar Frameworks
Source: ACS Omega. 2026 Jun 8;11(24):35846–55. doi: 10.1021/acsomega.6c02492 (PMC13295043; doi:10.1021/acsomega.6c02492)
Supplement: Supplementary file 1 [file ao6c02492_si_001.pdf]

Supporting Information

Synthesis and Structural Evolution of Zn-Ni ZIF Materials: From Cubic to Lamellar Frameworks

Iasmin Soares de Lima[1], Gabriel Alves da Silva[2], Lais Gomes Sanchez[2], Gabriel Iago dos Santos[2], Gilbert Bannach[2], Luiz G. Possato[2]*

[1]*Department of Physics and Meteorology, São Paulo State University (Unesp), School of Sciences, Av. Eng. Luiz Edmundo Carrijo Coube 14-01, Bauru, SP 17033-360, Brazil*

[2]*Department of Chemistry, São Paulo State University (Unesp), School of Sciences, Av. Eng. Luiz Edmundo Carrijo Coube 14-01, Bauru, SP 17033-360, Brazil*

*Corresponding author: gustavo.possato@unesp.br

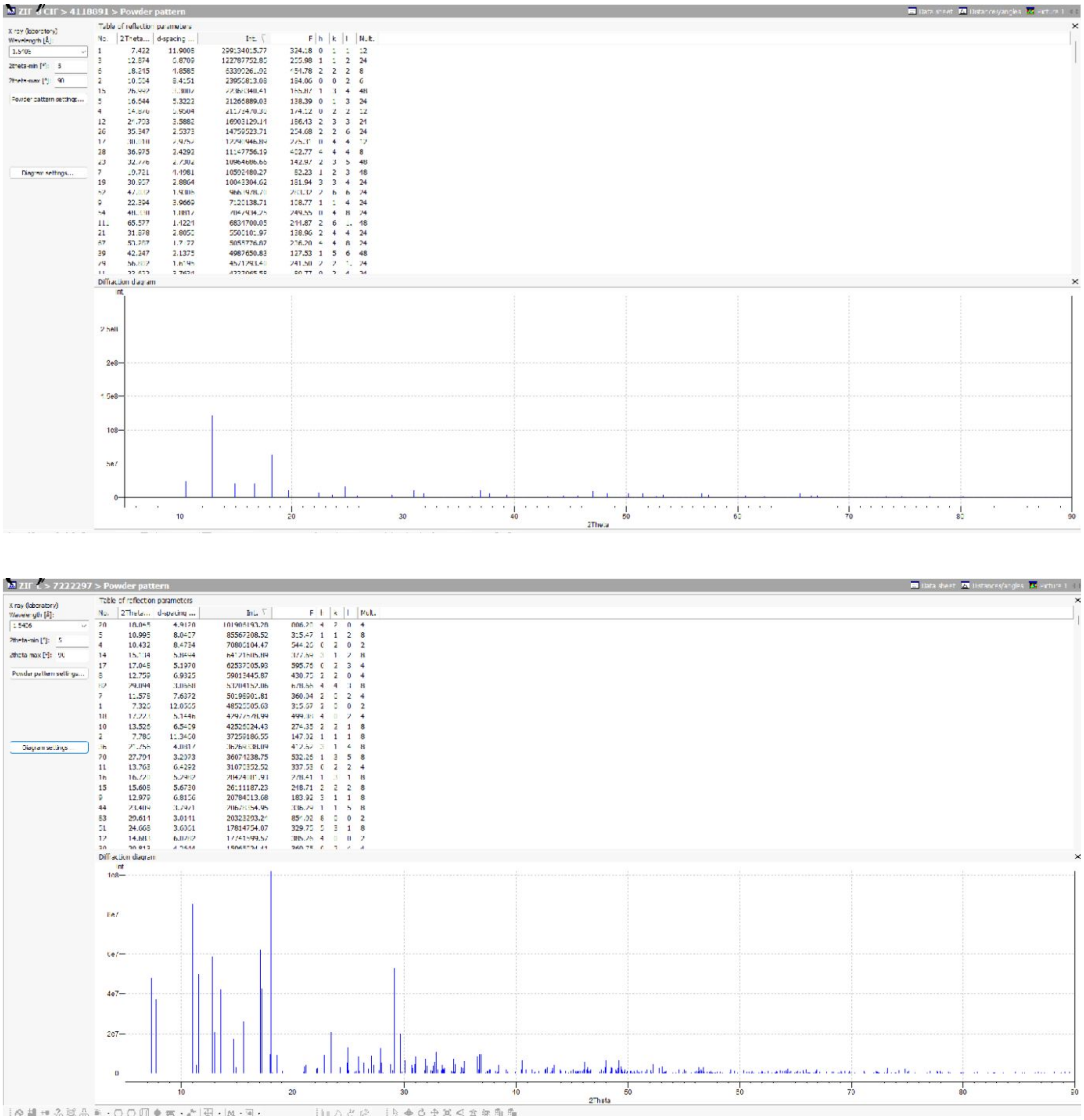

Figure S1. Reference X-ray diffraction (XRD) patterns of ZIF-8 (SOD topology) and ZIF-L (layered structure) used for phase identification and comparison with the $Zn_{1-x}Ni_x$ samples. The distinct diffraction features of each structure highlight the differences in topology and dimensionality between the cubic ZIF-8 and lamellar ZIF-L frameworks.

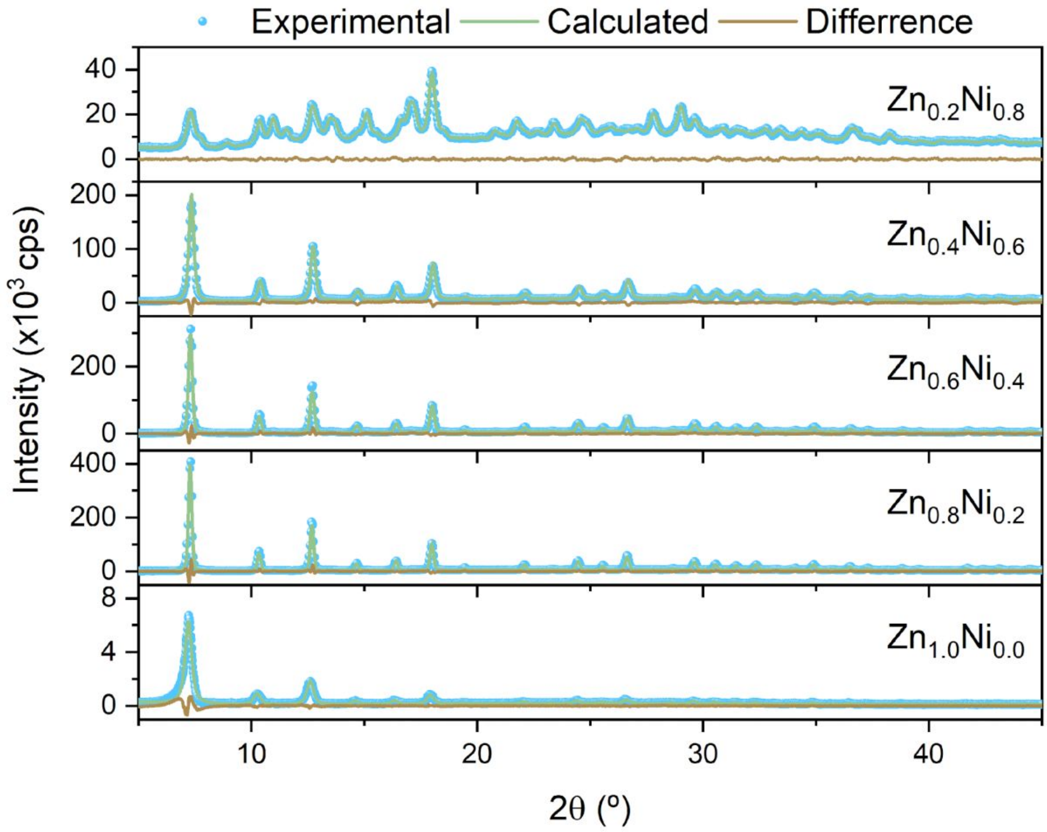

Figure S2. Rietveld refinement profiles of $Zn_{1-x}Ni_x$ (x = 0-0.8). Experimental (blue dots), calculated (green line), and difference (brown line) profiles are shown for each composition.

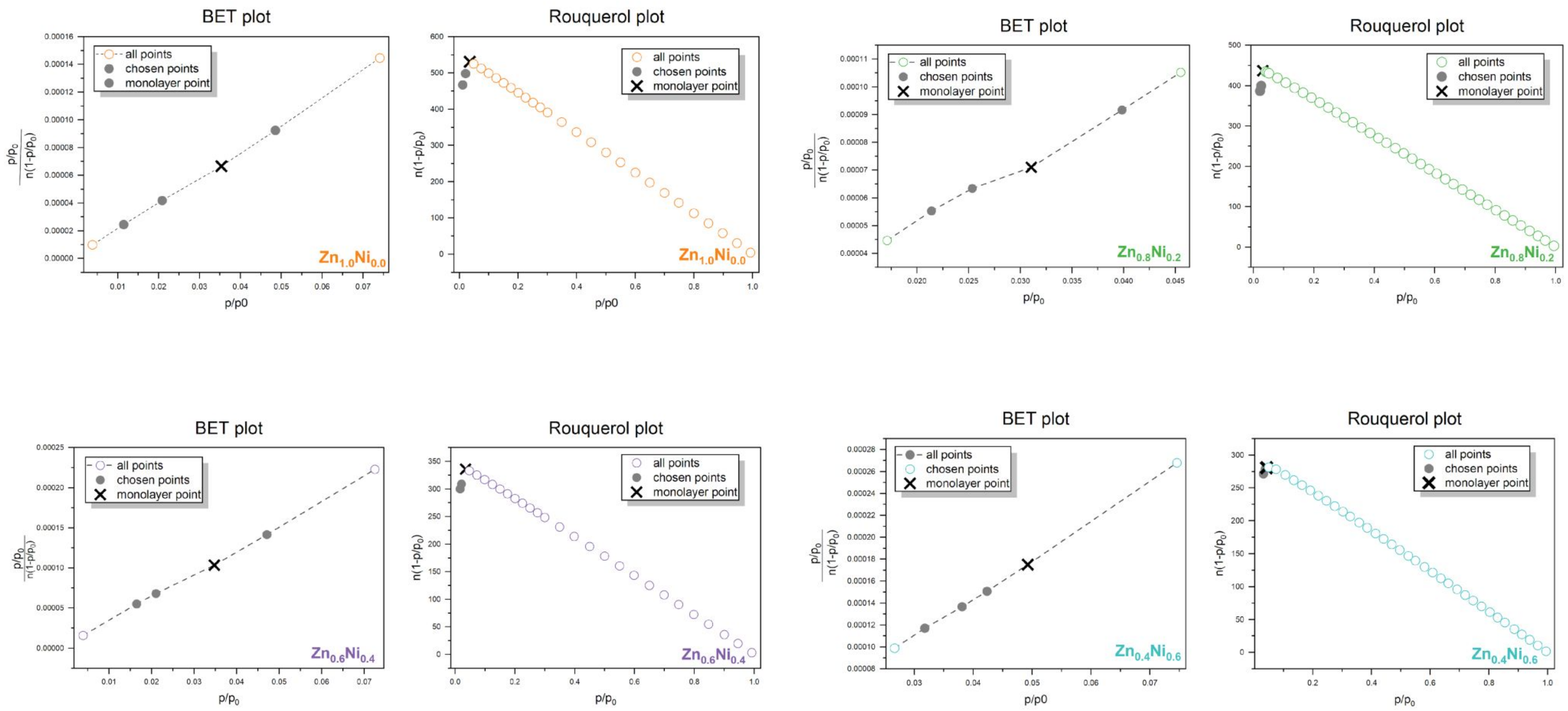
BET plot
Rouquerol plot
all points
chosen points
monolayer point
p/p0
n(1-p/p0)
Zn1.0Ni0.0
Zn0.8Ni0.2
Zn0.6Ni0.4
Zn0.4Ni0.6

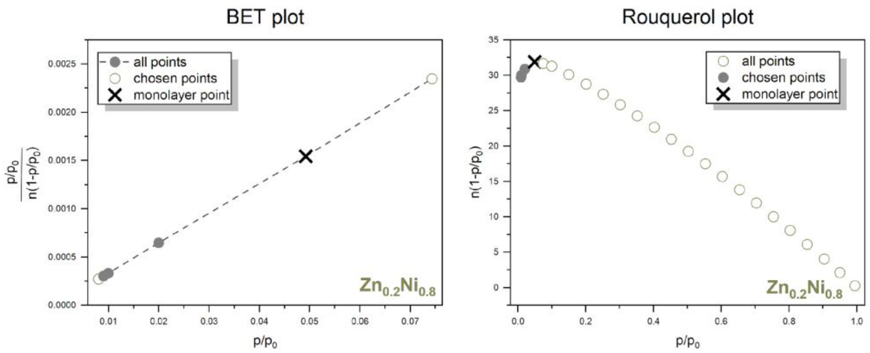

Figure S3. BET and Rouquerol plots used for the determination of the apparent surface area of $Zn_{1-x}Ni_x$ samples. The selected linear region (chosen points) satisfies the Rouquerol consistency criteria, ensuring the appropriate application of the BET model to microporous ZIF materials. The monolayer capacity point is also indicated.

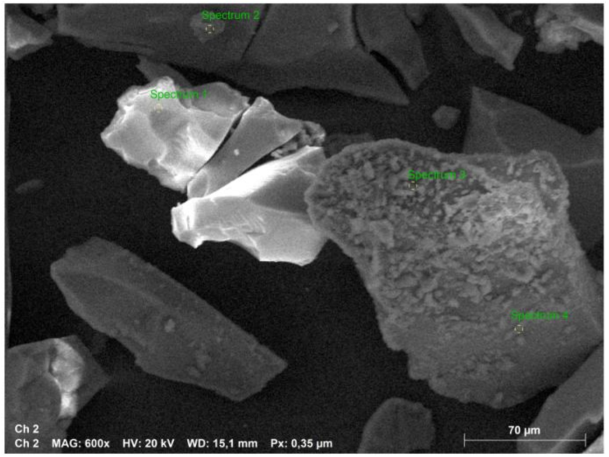

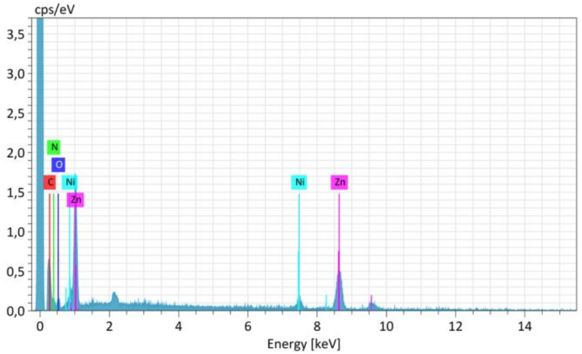

Spectrum 1

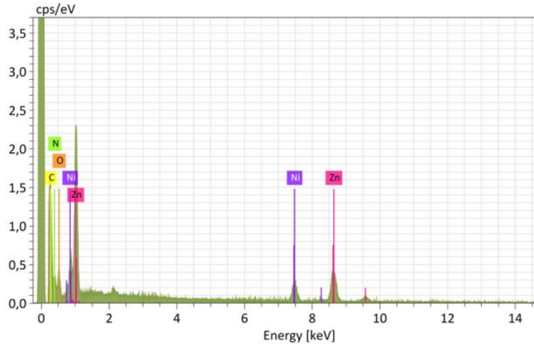

Spectrum 2

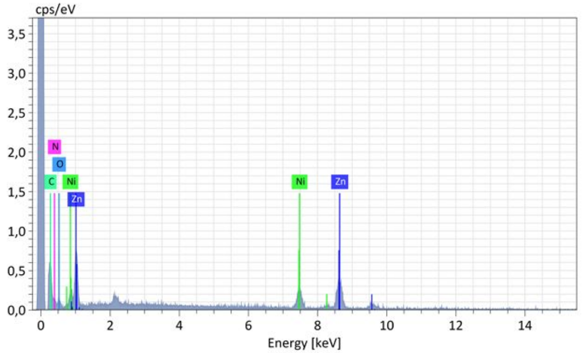

Spectrum 3

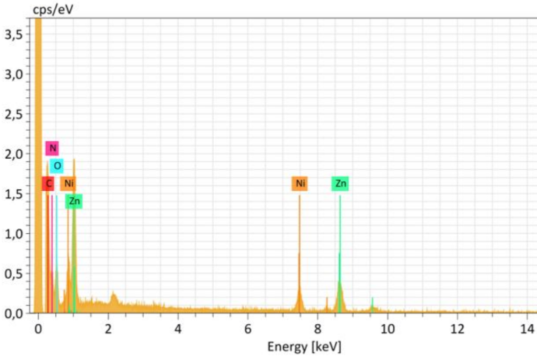

Spectrum 4

Figure S4. SEM micrograph and corresponding EDS spectra of the $Zn_{0.2}Ni_{0.8}$ sample. EDS spectra (Spectra 1-4) collected from distinct regions confirm the presence of Zn, Ni, C, and N, indicating homogeneous distribution of both metal ions within the framework. No additional elements were detected, confirming the chemical purity of the samples.
